# Prolonged Zika virus viremia in a patient with Guillain-Barré syndrome in Trinidad and Tobago

**DOI:** 10.26633/RPSP.2017.136

**Published:** 2017-09-29

**Authors:** Gabriel Gonzalez-Escobar, Anne Marie Valadere, Rosmond Adams, Karen Polson-Edwards, Avery Q.J. Hinds, Akenath Misir, C. James Hospedales

**Affiliations:** 1 Caribbean Public Health Agency (CARPHA) Port of Spain Trinidad and Tobago Caribbean Public Health Agency (CARPHA), Port of Spain, Trinidad and Tobago.; 2 Ministry of Health Ministry of Health Port of Spain Trinidad and Tobago Ministry of Health, Port of Spain, Trinidad and Tobago.

**Keywords:** Guillain-Barré syndrome;, polymerase chain reaction;, Zika virus;, Trinidad and Tobago., Síndrome de Guillain-Barré;, reacción en cadena de la polimerasa;, virus Zika;, Trinidad y Tobago., Síndrome de Guillain-Barré;, reação em cadeia da polimerase;, virus Zika;, Trinidad e Tobago.

## Abstract

*An emerging mosquito-borne flavivirus, Zika virus (ZIKV) is a significant public health concern because of the syndromes associated with the infection. In addition, ZIKV is considered a major problem due to large-scale spread of the disease and the possible clinical complications for the central nervous system, especially Guillain-Barré syndrome (GBS) and microcephaly. Since the introduction of ZIKV in the Caribbean, molecular detection of the viral RNA has been utilized as a more specific and sensitive approach to demonstrating acute infection. However, it is generally accepted that the virus has a short viremic period, generally less than 5 days. Serologic testing has the inconvenience of strong cross-reactivity among flaviviruses, such as dengue and yellow fever. As part of the laboratory surveillance activities for Zika and other arboviruses at the Caribbean Public Health Agency, in 2016 a sample from a male who was clinically diagnosed with GBS tested positive for Zika virus by real-time polymerase chain reaction (rRT-PCR). The serum sample had been taken on day 21 after the onset of symptoms. The case had initially been characterized as a typical ZIKV infection (mild fever with a generalized maculopapular rash). Later, weakness of limbs and other peripheral neurological symptoms appeared. Enzyme-linked immunoassay (ELISA) showed that the sample was negative for IgM antibodies against Zika, Chikungunya, and dengue viruses. The plaque reduction neutralization test was positive for ZIKV. This indicated parallel development of viremia and immune response against ZIKV. Recent reports have demonstrated a longer duration of the viremia in ZIKV infections. However, our report is the first one that links the infection with extended viremia and the development in parallel of a GBS case*.

## RESUMEN

El virus del Zika (ZIKV), un flavivirus emergente transmitido por mosquitos, es una inquietud importante en el ámbito de la salud pública por los síndromes asociados con la infección. Además, el ZIKV se considera un problema acuciante debido a la propagación a gran escala de la enfermedad y a las posibles complicaciones clínicas en el sistema nervioso central, en concreto, el síndrome de Guillain-Barré y la microcefalia. Desde que el ZIKV se introdujera en el Caribe, la detección molecular del ARN viral ha sido el método más específico y sensible utilizado para comprobar una infección aguda. Sin embargo, se cree que el virus tiene un período virémico corto, de menos de 5 días en general. La prueba serológica presenta el inconveniente de la fuerte reactividad cruzada entre los flavivirus, como el dengue y la fiebre amarilla. Como parte de las actividades de vigilancia de laboratorio para el Zika y otros arbovirus del Agencia Caribena de Salud Publica, en el 2016 la muestra de un hombre diagnosticado con la enfermedad de Guillain Barré dio positiva para el virus del Zika por medio de una reacción en cadena de la polimerasa en tiempo real (rRT-RCP). La muestra de suero se había tomado en el día 21 después de la aparición de los síntomas. En un principio, el caso se calificó como una infección por ZIKV típica (fiebre leve con una erupción maculopapular generalizada). Posteriormente, apareció la debilidad de los miembros y otros síntomas neurológicos periféricos. La prueba de inmunoadsorción enzimática (ELISA) fue negativa para los anticuerpos de tipo IgM contra los virus del Zika, el chikunguña y el dengue. La prueba de neutralización por reducción del número de placas dio positivo para el ZIKV, lo que probó que paralelamente se había desarrollado una respuesta virémica e inmune contra el ZIKV. En informes recientes se ha demostrado que en las infecciones de ZIKV el periodo virémico es más largo. Sin embargo, nuestro informe es de los primeros que relaciona un periodo virémico prolongado con el desarrollo paralelo del síndrome de Guillain-Barré.

## RESUMO

O vírus zika é um flavivírus emergente transmitido por mosquitos e tem sido motivo de grande preocupação em saúde pública por causa das síndromes associadas à infecção. É também considerado um importante problema devido à propagação em grande escala e possíveis complicações clínicas no sistema nervoso central decorrentes da infecção, sobretudo síndrome de Guillain-Barré e microcefalia. Desde a introdução do vírus zika no Caribe, a detecção molecular do RNA viral tem sido usada como método mais específico e sensível para demonstrar infecção aguda. Porém, admite-se em geral que o vírus tem um curto período virêmico, inferior a 5 dias. O teste sorológico tem o inconveniente de produzir intensa reação cruzada com outros flavivírus, como os vírus da dengue e febre amarela. Como parte da vigilância laboratorial do vírus zika e outros arbovírus pela Agência de Saúde Pública do Caribe, em 2016, foi examinada uma amostra de um paciente do sexo masculino com diagnóstico clínico de síndrome de Guillain-Barré e o resultado foi positivo para o vírus zika com a técnica de reação em cadeia da polimerase em tempo real. A amostra sérica havia sido coletada no dia 21 após o início dos sintomas. O caso foi inicialmente descrito como infecção típica pelo vírus zika (febre baixa com erupção cutânea maculopapular generalizada) e, posteriormente, o paciente apresentou fraqueza dos membros e outros sintomas neurológicos periféricos. A amostra foi testada com a técnica de imunoensaio enzimático (ELISA) e foi negativa para anticorpos da classe IgM contra o zika vírus, vírus chikungunya e vírus da dengue. O teste de neutralização por redução de placas foi positivo para o vírus zika, indicando aumento em paralelo da viremia e resposta imunológica ao vírus. Informes recentes têm demonstrado viremia de duração mais prolongada em infecções por vírus zika. Porém, este é o primeiro relato que associa a infecção com viremia prolongada ao surgimento em paralelo da síndrome de Guillain-Barré.

Zika virus (ZIKV) is an arthropod-borne virus in the genus *Flavivirus*, family *Flaviviridae* (1). The genus *Flavivirus* encompasses many relevant human pathogens, including yellow fever,dengue, West Nile Fever, Japanese encephalitis, and tick-borne encephalitis viruses. ZIKV was first isolated in 1947 from a febrile macaque monkey in Uganda (2) and was subsequently isolated from *Aedes africanus* mosquitoes (3).

ZIKV disease is usually a mild and self-limiting illness associated with such symptoms as fever, maculopapular and pruritic rash, arthralgia, and nonpurulent conjunctivitis (4). Since theemergence of the disease, many reports have connected Zika virus infection with Guillain-Barré syndrome (GBS), as was demonstrated previously for other flaviviruses (5).

GBS cases temporally associated with short-term ZIKV infection (viremia less than 5 to 7 days after the onset of symptoms) have been reported in French Polynesia (6); Brazil, Colombia, the Dominican Republic, El Salvador,Honduras, Suriname, and Venezuela (7); and Haiti (8). We here report the finding of a case of GBS with prolonged ZIKV viremia, in a patient from the Caribbean island country of Trinidad and Tobago. Both molecular detection of the target viral RNA and serologic characterization of the serum were used as diagnostic approaches.

## EVIDENCE OF PROLONGED ZIKA VIRUS VIREMIA IN A PATIENT WITH GUILLAINBARRÉ SYNDROME

On 10 August 2016, a 29-year-old man visited his private practitioner in Tunapuna, a city that is in the north of Trinidad and Tobago, about 15 km from the capital city of Port of Spain. The initial clinical symptoms were characterized as nonquantified mild fever, headache, and malaise. No respiratory or neurological symptoms were present at that time, and the patient was sent home with symptomatic treatment. No specimens from any source were taken during this first visit.

Febrile peaks continued appearing during the days after the onset of symptoms. A generalized maculopapular rash and a tingling and weakness of lower limbs appeared on 16 August (day 7 after the onset of symptoms). However, following a medical examination by the same private practitioner, the patient was not hospitalized.

Rash completely disappeared on 20 August (day 11 after the onset of symptoms), but weakness of lower limbs (associated with walking instability) progressed to the upper limbs on 30 August (day 21), when the patient was sent by the private practitioner for neurological evaluation and hospitalization at the Port of Spain General Hospital.

After the patient was hospitalized, the clinical examination revealed a moderate to severe impairment of muscle strength. The patient was clinically diagnosed with Guillain-Barré syndrome, and therapeutic schemes based on the administration of intravenous immunoglobulin were initiated.

A blood specimen was taken that same day (August 30, day 21after the onset of symptoms) and sent to the Trinidad Public Health Laboratory for virologic/serological investigation. (This was the only blood specimen taken from the patient during the illness.) That serum sample was then sent on 5 September for laboratory investigation at the Laboratory of Virology of the Caribbean Public Health Agency (CARPHA), in the city of Port of Spain.

Enzyme-linked immunoassay (ELISA) demonstrated the absence of IgM antibodies against dengue virus (Dengue Virus IgM Capture DxSelect, Focus Diagnostics, Cypress, California, United States of America), Chikungunya virus (Anti-Chikungunya Virus ELISA IgM, EUROIMMUN AG, Lübeck, Schleswig-Holstein, Germany), and ZIKV (Anti-Zika virus ELISA IgM, EUROIMMUN AG, Lübeck, Schleswig-Holstein, Germany). Testing of viral RNA for dengue virus (DENV), Chikungunya virus (CHIKV), and ZIKV was done using Trioplex Real-time RT-PCR assay (rRTPCR) (Centers for Disease Control and Prevention (CDC), Dengue Branch, San Juan, Puerto Rico), following the CDC’s instructions for its use. Viral RNA detection was found negative for DENV and CHIKV, and positive for ZIKV. The crossing threshold (Ct) value found for the sample was 34.46 (positive below 38).

On 12 October 2016, an aliquot of the original serum sample was sent for laboratory confirmation to the Division of Vector-Borne Diseases, which is part of the CDC’s National Center for Infectious Diseases, in Fort Collins, Colorado, United States. The arboviral serology report on 17 November indicated evidence of infection with a flavivirus, according to neutralizing antibody testing, with a plaque reduction neutralization test (PRNT) titer > 1 280 for ZIKV (positive control titer > 320).

As of May 2017, the patient was still reporting a mild persistence of weakness of both lower and upper limbs (personal communication from patient, May 2017).

## LIMITATIONS

This study has some limitations, including the absence of early and successive (serial) samples. These samples should have been taken between days 1 to 5 after the onset of symptoms. Moreover, it also could have been useful to take some samples at different intervals (for instance, 7, 10, 15, 20, and 30 days after the onset). With all these specimens, the kinetics of the viral progression in blood could have been determined. In addition, serial sampling could have been very useful for establishing the relation between the initial symptoms and the suspected ZIKV infection. Moreover,additional samples could have also demonstrated the clearance of the virus in blood and/or other body fluids (e.g., urine). On the other hand, despite the fact that the quantification of viral load in this sample was relative instead of absolute, the Ct value reported from the rRTPCR assay is an indication that the target RNA was present in the sample. No additional samples from this patient were obtained and, therefore, a follow-up determination of the relative amount of target RNA could not be performed.

## DISCUSSION

In this study, a male was clinically diagnosed with an infection produced by ZIKV, and he later developed GBS. The patient is a resident of Tunapuna, an urban area with a large population in the northern part of the island of Trinidad. Other cases of ZIKV infection had been found in the area, and there was a relatively low incidence of other arbovirus infections, such as DENV and CHIKV, at that time.

The ZIKV case was clinically well characterized as a mild ZIKV infection with fever and maculopapular rash, with no criteria for hospitalization at the beginning of the illness. On day 7 after the onset of symptoms, neurologic symptoms were evident, which progressed from the lower limbs to the upper limbs on day 21, when the patient was hospitalized and a blood specimen was taken. According to the algorithm for detection of ZIKV infections at the CARPHA’s Laboratory of Virology, such specimens are tested within 7 days after the onset of symptoms. However, in some cases, such as pregnant women or GBS patients, the laboratory staff may decide (generally for research purposes) to include the samples for diagnosis, even though the samples do not match with the algorithms for the detection of ZIKV.

Amplification of Zika virus RNA in serum samples is the most specific and sensitive diagnostic approach available, and it must be performed during the acute phase (viremic period) of the illness (up to 7 days from the onset of symptoms). In March 2016, the Pan American Health Organization (PAHO) developed case definitions and surveillance guidance for Zika virus disease and associated complications that clearly indicated a characteristic transient viremic period during ZIKV infections (9).

Abundant published evidence shows that negative amplifications have been reported for patients whose samples were tested at 6 or more days after the onset of symptoms, indicating a viremic period as brief as 5 days (10). This agrees with other observations, in which viremia was detected when symptoms were present, but not afterward (11). In contrast, prolonged Zika virus RNA has been detected in serum from symptomatic pregnant women from 35 to 53 days post-exposure (12, 13). Additionally, viremia is very often low level, making viral isolation from clinical samples difficult (10).

In a case-control study (6), acute Zika virus infection, as confirmed by a positive RT-PCR result, was observed for all patients in one of the control groups, but for none of the 41 patients tested in the GBS group. According to the authors, this corroborated some clinical observations (notably the absence of fever), suggesting that the patients in the GBS group were no longer viremic at admission. In other words, viral nucleic acid was not amplified at the time when the GBS symptoms appeared. Moreover, in a recently published preliminary report (14), the authors provided evidence that ZIKV is present in serum for a longer period than expected for other flaviviruses; the relation of this finding with GBS cases was not part of that study.

Our target sample was taken on day 21 from the onset of fever and day 15 from the onset of rash. By that time, according to our crossing threshold result (Ct = 34.46) in the rRT-PCR assay, the sample contained moderate amounts of target nucleic acid (Zika virus RNA) (considering a value of < 29 as a strong positive, between 30 and 37 as a moderate positive, and 38 to 40 as a weak or negative reaction). Given the high sensitivity and specificity of the rRT-PCR assay and the clinical and epidemiological characterization of this case, it appears that this infection with ZIKV was strongly associated with GBS in the patient. It also indicates that the viremia persisted beyond the time reported previously for Zika and other flaviviruses.

Isolation of ZIKV to demonstrate infectivity could not be performed at the CARPHA laboratory. Nevertheless, the resultant amplification of the target viral RNA is strong evidence that the nucleic acid was extracted from intact (and therefore, infective) viral particles. Free viral RNA in serum is unlikely due to ribonucleases, and remnant virus or non-infective particles are not described for most flaviviruses, at least in the context of persistent infections.

In serology testing (ELISA IgM), the sample was negative for DENV, CHIKV, and ZIKV. Many of the commercial kits for detection of IgM antibodies against ZIKV have low sensitivity, according to one study (15) and a recently circulated CARPHA internal report. This may explain a possible false-negative result, although a failure of IgM response cannot be completely ruled out.

An interesting question at this point is why ZIKV clearance in this patient was delayed and how the immune response could have been implied. One possible explanation is the existence of an unknown or poorly defined immune response mechanism in the course of a flavivirus infection. In fact, a 2014 study demonstrated that West Nile Virus–specific immune responses are severely impaired and delayed in diabetic mice (16), suppressing key antiviral defense responses such as those elicited by IFN-α, IgM, and IgG antibodies. In the same study, suppressed clearance of virus was observed in serum, peripheral tissues, and the brain, thus explaining the persistence of West Nile Virus. In our study there is no evidence that a known or unknown factor might have been responsible for the negative ELISA results and the prolonged viremia in the patient. However, other underlying and still not-defined mechanisms might be implicit. An impaired IgM response is one hypothesis.

The plaque reduction neutralization test (PRNT), an assay used to quantify the titer of neutralizing antibodies, showed neutralization of ZIKV at > 1 280 serum dilution. The neutralization observed is a consequence of the presence of neutralizing antibodies (mostly IgG) against ZIKV. Due to the limited amount of sample available, the lack of sensitivity, and the intense cross-reactivity among flaviviruses, demonstration of IgG antibodies against ZIKV and other flaviviruses was not included in this study. Nevertheless, it is logical that other anti-flavivirus IgG antibodies were present in the patient’s serum. That is especially true given that these viruses (such as DENV) have been endemic in the tropics for many years ago and that yellow fever vaccine is regularly applied in Trinidad and Tobago.

A mechanism known as antibody-dependent enhancement might be involved, in which IgG antibodies against viral envelope proteins resulting from a prior infection bind to virus particles of a subsequent infection. This leads to enhanced replication, and potentially more severe illness (17). Thus, persistent viremia that produces GBS could be related with that antibody-dependent enhancement mechanism. Evidence from in vitro experiments suggests cross-reactivity between DENV and ZIKV antibody responses and antibody-dependent enhancement of ZIKV by DENV antibodies (17–19). More research is needed on ZIKV persistent viremia and its relationship with immune response. Additionally, causes of further development of GBS must be investigated.

Many aspects of the ZIKV infection and pathogenesis in humans remain unclear. One remarkable example is whether amplification of viral RNA is related with effective infectious particles. Another striking case is the mechanisms responsible for the appearance of neurologic complications during the ZIKV exposure. With regards to the neurologic damage, one explanation indicates a possible antibody-response mechanism against specific antigens on the ZIKV particles. Another proposed explanation is direct damage of peripheral nerves produced by the virus itself (4, 13). Persistent ZIKV viremia, demonstrated as RNA presence in serum or blood, could be involved in those proposed mechanisms. Long-term ZIKV nucleic acid amplification has recently been related with congenital central nervous system problems, mostly microcephaly and brain development alterations (20). It should be established whether nervous tissue damage is a direct consequence of ZIKV infection. The molecular mechanisms implicated in this damage should also be assessed.

The data presented in this study provide evidence to support changing how we distinguish between an acute ZIKV infection and a nonacute one. Epidemiological surveillance systems, especially those for neurological disorders (for instance, acute flaccid paralysis surveillance for discarding polio virus infection), could be direct beneficiaries of a change in the parameters for laboratory detection (algorithms) of agents implicated in neurological illnesses (as with ZIKV and GBS).

There is growing evidence on the differences among flaviviruses in terms of the early stages of the illnesses, the clinical and epidemiological findings, and the duration of the viremia. The data presented in this article indicate that it is possible to determine an etiological diagnosis of a particular neurological disorder by modifying some laboratory algorithms, thus extending the proposed time for detection of some viral agents, including ZIKV.

To our knowledge, this is the first report that directly links long-term persistence of ZIKV infection with a non-congenital neurologic syndrome. We here demonstrated the concurrence of Zika virus viremia with the development of a case of Guillain-Barré syndrome. Further investigations are needed on the epidemiological relevance of our findings, the duration of the viremia, and the syndromes associated with ZIKV infection.

### Acknowledgments.

The authors would like to thank the staff of the Caribbean Public Health Agency (CARPHA), the Ministry of Health of the Republic of Trinidad and Tobago, the patient, and the physician for their input and contributions to this paper.

### Disclaimer.

The authors hold sole responsibility for the views expressed in the manuscript, which may not necessarily reflect the opinion or policy of the Caribbean Public Health Agency (Port of Spain, Trinidad and Tobago), the Ministry of Health of the Republic of Trinidad and Tobago (Port of Spain), the *RPSP/PAJPH*, or PAHO.
